# Anxiety among Doctors during COVID-19 Pandemic in Secondary and Tertiary Care Hospitals

**DOI:** 10.12669/pjms.36.6.3113

**Published:** 2020

**Authors:** Syed Riazul Hasan, Zeeshan Hamid, Muhammad Talha Jawaid, Rashida Kaizar Ali

**Affiliations:** 1 Dr. Syed Riazul Hasan, FCPS. Head of Department, Department of Internal Medicine, Sindh Government Qatar Hospital, Karachi, Pakistan; 2 Dr. Zeeshan Hamid, MBBS. Resident Medical Officer, Department of Internal Medicine, Sindh Government Qatar Hospital, Karachi, Pakistan; 3 Dr. Muhammad Talha Jawaid, MBBS. Post-graduate Trainee, Department of Internal Medicine, Sindh Government Qatar Hospital, Karachi, Pakistan; 4 Dr. Rashida Kaizar Ali, MBBS. Post-graduate Trainee, Department of Internal Medicine, Sindh Government Qatar Hospital, Karachi, Pakistan

**Keywords:** COVID-19, Anxiety, GAD-7, Doctors, Pandemic

## Abstract

**Objective:**

To assess the level of anxiety among doctors during COVID-19 pandemic and the associated risk factors.

**Methods:**

This cross-sectional study was conducted from 30th April to 16th May, 2020 in Karachi, Pakistan. The data was collected via an online web-based questionnaire. Questionnaire was used to assess anxiety level using GAD-7 scale among health-care professionals and the risk factors playing role in it.

**Results:**

One hundred and fifty-one doctors participated in our study. Out of these 151 participants, 69 (45.7%) had mild, 22 (14.6%) had moderate, and 5 (3.3%) had severe symptoms of anxiety, whereas the remaining 55 (36.4%) had no anxiety according to GAD-7 scale. The median [interquartile range (IQR)] GAD-7 scale scores are 6.0 [3.00-9.00]. Females showed more severe degrees of measurement of anxiety symptoms than males. Doctors dealing with COVID-19 patients showed higher level of anxiety as compared to the doctors who were not dealing with COVID-19 patients, having a significant difference (U = 9.697, p = 0.008). One hundred and forty-one (93.4%) participants were concerned about being exposed to COVID-19 at work and 112 (74.2 %) thought they have inadequate protective equipment for safety.

**Conclusions:**

During COVID-19 pandemic, doctors exhibited different grades of anxiety. In order for healthcare workers to perform to the best of their capability, certain guidelines and interventions are needed.

## INTRODUCTION

COVID-19, that took the world by storm, emerged as a worldwide health care crisis, resulting in the first pandemic of the 21st century. It first was reported in December 2019 to the World Health Organization as a cluster of cases of pneumonia in the city of Wuhan, China which was later identified as a novel coronavirus.^1^ Soon it spread to the other countries, and by 9th of August 2020, the total number of confirmed infected cases has crossed over 2.7 million,^2^ and the total death toll has crossed 7,22,285.^2^

In Pakistan, the first confirmed case was reported by the Ministry of Health, Government of Pakistan on 26^th^ February, 2020 in the city of Karachi, Sindh province.^3^ By 10^th^ of August, 2020, the total number of confirmed cases in Pakistan had reached to 284,660 with 6,097 deaths reported.^4^ The ever-increasing number of cases has not only created a panic among the general population but has also increased the workload on the healthcare system resulting in an add-on pressure on the health care professionals and the personnel associated with health care system including the health care administration. The overwhelming burden of the pandemic has resulted in not only the physical but psychological pressure on health care workers. This has previously been seen as deteriorating psychological impact on health care workers during the SARS outbreak in 2013.^5^

Knowing that this health crisis could last months, it is also essential that the health care professionals and providers are well taken care of not just physically but psychologically for them to be able to perform to their full potential. In doing that, we conducted a survey to assess the level of anxiety among health care providers and look for the factors that might be responsible for it. The study would help in building a foundation for certain guidelines to be made for the better care of the personnel associated with health care by the institution on a limited scale and by national health governing bodies on a larger scale in Pakistan.

## METHODS

This cross-sectional study was conducted between 30th April, 2020 and 16th May, 2020. A total of 151 doctors working in both public and private sector hospitals in the city of Karachi to participate in this survey after taking informed consent. Study protocol was approved for data collection by the Medical Superintendent of Sindh Government Qatar Hospital, Karachi.

### Ethical Approval

(Ref No: SGQH/2929, Dated: 18-06-2020).

The data was collected via an online web-based questionnaire. The questionnaire included a mandatory pre-requisite informed consent. A psychiatrist working at Sindh Government Qatar Hospital, Karachi was consulted who provided his valuable input on the questionnaire. The questionnaire consisted of three parts: A) Basic demographic data which included age, gender, marital status, education level (bachelor’s or master’s), working as (a doctor or an administrative official), working in capacity of (administrative official not seeing patients- LEVEL ONE, health care professional seeing patients other than COVID-19- LEVEL TWO, health care professionals seeing patients of suspected or confirmed COVID-19 in Emergency Department or Isolation Ward- LEVEL THREE). B) A GAD-7 questionnaire was used to assess anxiety. GAD-7 as proposed by Spitzer RL et al.^6^ consists of 7 items is helpful as a screening tool for anxiety and assessing its severity in clinical practice.^6^ Participants were asked how often during the last two weeks; they faced the mentioned symptoms. The responses were recorded as “not at all”, “several days”, “more than half the days”, and “nearly every day”, and subsequent scores were given as 0, 1, 2, and 3 respectively. The total score for the seven items ranges from 0 to 21. At a score of 10 or more, sensitivity and specificity exceed 80%.^7^ The scores were assessed according to the following criteria:^6^,^8^,^9^ (5–9): mild anxiety, (10–14): moderate anxiety (15–21): severe anxiety. C) Risk factors associated with that anxiety were addressed that might be playing role in precipitating the anxiety including being exposed to COVID-19, having access to protective equipment, having access to safety precautions at workplace, concerns about family members, feeling exhaustion at work and having necessary knowledge regarding precautionary measures and COVID-19.

### Statistical Analysis

Data was analyzed using IBM SPSS Statistics software (version 26). For categorical variables, the number of cases and percentages were used. The GAD-7 score was not normally distributed and hence is presented as medians with interquartile ranges (IQRs). The nonparametric tests Mann-Whitney U Test and Kruskal-Wallis Test were performed for comparing non-normally distributed continuous dependent variables with categorical independent variables. The level of significance was set as p < 0.05.

## RESULTS

Out of a total of 151 doctors who participated, a total of 85 (56.3%) were Females (Table-I). Mean age was 29 (±7.28) years, out of which majority 89 (58.9%) were of age less than 29 years. A total of 76 (50.3%) were not married. The other demographic data is shown in (Table-I).

**Table-I:** Demographic Characteristics of Participants.

Characteristic	N (%)		Total N
Gender	Male	66 (43.7%)	151
	Female	85 (56.3%)	
Age	18-29 years	89 (58.9)	151
	30-39 years	50 (33.1%)	
	40-49 years	10 (6.6%)	
	Above 50 years	2 (1.3%)	
Marital Status	Single	76 (50.3%)	151
	Married	75 (49.7%)	
Education	MBBS	115 (76.2%)	151
	Post-graduation (FCPS)	36 (23.8%)	
Institution	Public sector hospital	106 (70.2%)	151
	Private sector hospital	45 (29.8%)	
Working In Capacity of	Doctors working as administrative officials not dealing with patients directly (Level-1, at low risk)	20 (13.2%)	151
	Doctors seeing patients other than COVID-19 (Level-2, at medium risk)	90 (59.6%)	
	Doctors seeing patients of suspected or confirmed COVID-19 in Emergency Department or Isolation Ward (Level-3, at high risk)	41 (27.2%)	

Out of 151 participants, 69 (45.7%) had mild, 22 (14.6%) had moderate, and 5 (3.3%) had severe symptoms of anxiety, whereas the remaining 55 (36.4%) had GAD-7 scale score of less than 5, which is considered normal or healthy level of anxiety. The original scores of GAD-7 tool were not normally distributed and so are presented as Medians with interquartile ranges (IQRs). Out of a total of 151 participants, the median [interquartile range (IQR)] GAD-7 scale scores is 6.0 [3.00-9.00].

A Mann-Whitney U test showed that there was a significant difference (U = 4004, p < 0.001) between the GAD-7 scores for males compared to females. Females showed more severe degrees of measurement of anxiety symptoms than males, as median [interquartile range (IQR)] GAD-7 scale scores among males versus females: 4.0 [2.0-7.0] versus 8.0 [5.0-9.0]; [p<0.001]. The comparison of the distribution of GAD-7 scores between the different categories of marital status, education, institution, and level of exposure is shown in (Table-II).

**Table-II:** Gad-7 Anxiety Scores Assessment and Comparison between Groups.

Components		Total Participants N=151(%)	Median and Interquartile Range (IQR) for GAD-7 Score	Mann-Whitney U test and P value*	Statistically Significant (P< .05) Interpretation
Level of Anxiety	Normal	55 (36.4%)	6.0 [3.0-9.0]	---	---
	Mild	69 (45.7%)			
	Moderate	22 (14.6%)			
	Severe	5 (3.3%)			
Gender Difference	Male	66 (43.7%)	4.0 [2.0-7.0]	(U = 4004, P < 0.001)	Females showed more severe degrees of measurement of anxiety symptoms than males.
	Female	85 (56.3%)	8.0 [5.0-9.0]		
Marital Status Difference	Single	76 (50.3%)	6.0 [3.0-9.0]	(U = 3013, P = .543)	There was not a significant difference between the GAD-7 scores for single compared to married participants.
	Married	75 (49.7%)	7.0 [4.0-9.0]		
Education Difference	MBBS	115 (76.2%)	7.00 [4.0-9.0]	(U=1948.5, P = .594)	There was not a significant difference between the GAD-7 scores for doctors having only MBBS degree compared to doctors having Post-graduation degree.
	Postgraduation	36 (23.8%)	5.50 [3.0-9.0]		
Institution Difference	Public sector	106 (70.2%)	7.00 [3.0-9.0]	(U=2149.0, P = .335)	There was not a significant difference between the GAD-7 scores for doctors working in public compared to private sector.
	Private sector	45 (29.8%)	5.00 [4.0-8.0]		
High Risk Exposure to Medium and Low Risk Exposure Difference	Level-3	41 (27.2%)	8.0 [4.0-11.0]	(U = 9.697, P = .008)*	LEVEL-3 (at high risk exposure) doctors dealing with suspected or confirmed COVID-19 patients showed more severe anxiety symptoms than doctors not dealing with COVID-19 patients directly.
	Level-2	90 (59.6%)	6.0 [2.0-8.0]		
	Level-1	20 (13.2%)	5.0 [3.5-7.5]		

* A Kruskal-Wallis Test was used for statistical difference between the GAD-7 scores for the group of LEVEL-3 (at high risk exposure) compared to the groups of LEVEL-2 (at medium risk exposure) and LEVEL-1 (at low risk exposure) doctors.

Out of a total of 151 participants, 45 (29.8%) had access to surgical mask; latex or plastic gloves only, 9 (6.0%) had access to surgical mask only, 2 (1.3%) had access to latex or plastic gloves only, 4 (2.6%) had no access to protective equipment, 17 (11.2%) had access to surgical mask; N-95 respirator; latex or plastic gloves; surgical cap; goggles; face shield; personal protective gown, 14 (9.3%) had access to surgical mask; N-95 respirator; latex or plastic gloves, the others (39.8%) had different combinations of equipment.

Out of a total of 151 participants, 141 (93.4%) were concerned about being exposed to COVID-19 at work. Twenty-two (14.6%) had infants less than one year of age at home, 56 (37.1%) had adults above 60 years of age, 40 (26.5%) had both infants and adults, and 33 (21.9%) did not have either infants or adults. The other possible factors precipitating anxiety are shown in (Table-III).

**Table-III:** Risk Factors Association

FACTORS		Total Participants N=151(%)	

	Yes	No	OPT Not to Answer
Concerned about being exposed to COVID-19 at work	141 (93.4%)	9 (1.0%)	1 (0.7%)
Inadequate protective equipment for safety	112 (74.2%)	37 (24.5%)	2 (1.3%)
Fear of taking the infection to loved ones at home	147 (97.4 %)	3 (2.0%)	1 (0.7%)
Doubtful about working in a pandemic	88 (58.3%)	56 (37.1%)	7 (4.6%)
Concerned about having access to testing facilities if they develop COVID-19 symptoms	95 (62.9%)	52 (34.4%)	4 (2.6%)
Concerned about being up-to-date of knowledge on dealing with COVID-19 patients	121 (80.1%)	27 (17.9%)	3 (2.0%)
Concerned if their organization will take care of their families in case they get infected with COVID-19 while working during a pandemic	107 (70.9%)	33 (21.9%)	11 (7.3%)
Concerned about being able to perform to the best of their ability if transferred to a new facility during pandemic	119 (78.8%)	24 (15.9%)	8 (5.3%)
Concerned about having access to soap with water and/or hand sanitizer at workplace	96 (63.6%)	54 (35.8%)	1 (0.7%)
Anticipation of feeling exhausted while working	113 (74.8%)	36 (23.8%)	2 (1.3%)
Fear of being isolated in case they develop COVID-19 symptoms	120 (79.5%)	29 (19.2%)	2 (1.3%)
National duty to work during a pandemic	112 (74.2%)	25 (16.6%)	14 (9.3%)

## DISCUSSION

The health care professionals in general, and the front-line health care workers dealing with COVID-19 patients directly in specific, are facing a situation that they were never prepared for. The physical over-burden along with the psychological impact of such a pandemic has resulted in increased anxiety among the health care workers. In our study, we assessed anxiety among doctors and the associated risk factors during COVID-19.

A cross-sectional study done in China where they addressed factors associated with mental health outcomes among health care workers, in which they used the 7-item Generalized Anxiety Disorder scale, showed that 44.6% participants had symptoms of anxiety.^7^ The cutoff score they used for detecting symptoms of anxiety using GAD-7 was 7 and participants who had scores greater than the cutoff threshold were characterized as having severe symptoms.^7^ In our study, we focused on anxiety and also used the 7-item Generalized Anxiety Disorder (GAD-7) for assessing anxiety.

In our study, out of a total of 151 participants, 69 (45.7%) had mild, 22 (14.6%) had moderate, and 5 (3.3%) had severe symptoms of anxiety.^6^,^8^,^9^ A total of 56.3% participants were predominantly females, which is comparable to the study done in China which also had a female (76.7%) predominance.^7^ The median [Interquartile range(IQR)] for GAD-7 scores in our study for males versus females: 4.0 [2.0-7.0] versus 8.0 [5.0-9.0] showing females had more severe degrees of measurement of anxiety symptoms than males, which was again similar to the study in China which had the median [Interquartile range(IQR)] for GAD-7 for males versus females: 2.0 [0-6.0] versus 4.0 [1.0-7.0].^7^ In our study, Level-3 (at high risk exposure) doctors who were dealing with suspected or confirmed COVID-19 patients had higher GAD-7 median scores compared to Level-2 (at medium risk exposure) and Level-1 (at low risk exposure) doctors, which was again similar to the study in China which showed frontline health care workers diagnosing and treating COVID-19 patients were associated with high risk of anxiety symptoms.^7^

Another study done in Singapore during COVID-19 pandemic in which they used Depression, Anxiety, and Stress Scales (DASS-21) to assess overall mental status showed that 14.5% participants screened positive for anxiety,^10^ whereas in our study, we used GAD-7 to assess anxiety and results showed 27 (17.9%) participants had moderate-severe anxiety. Another study done in Peshawar city of Pakistan during March-April 2020 which assessed the personal and professional impact of COVID-19 on health care professionals.^11^ It was a qualitative study and showed that the participants felt anxious, frustrated and stressed out. This study had participants mostly females (56.5%) similar to our study having females (56.3%) predominant.

While the COVID-19 situation in hand is unexpected, and perhaps not well prepared for, drastic measures need to be taken and ensured in order to maximize the workforce that is available. The first and foremost consideration in this regard is communicating with the health care workers by the organization, especially the hospital leadership along with professional psychologists. Actively communicating with the health care workers is the key in reducing anxiety. Out of a total of 151 participants, 121 (80.1%) were concerned about being up-to-date of knowledge on dealing with COVID-19 patients, 27 (17.9%) said they were up-to-date of knowledge on dealing with COVID-19 patients, and 3 (2.0%) opted not to answer. Special emphasis should be given on guidelines for protecting themselves, their family members, dealing with the patients, keeping up-to-date about knowledge regarding COVID-19, and any individual concerns.

The patients might not be suspected of having COVID-19, but they might be asymptomatic and yet transmitting the disease^12^, hence precautionary measures need to be taken. Letting the health care workers know that their safety is of priority focus for the organization. Overall, 112 (74.2 %) participants thought they have inadequate protective equipment for safety from COVID-19. Knowing that protective equipment is critically short in such times of crisis, health care professionals such as administrative officials should have access to face surgical masks at the least, doctors who deal with patients other than COVID-19 should have access to face surgical masks, plastic or latex gloves, and goggles (in case dealing with patients with possible respiratory illness)^13^, and doctors who deal with suspected or confirmed COVID-19 patients should have access to N-95 respirator, latex gloves, surgical cap, goggles or face shield along with personal protective gown.

Along with the protective gear, the health care workers need access to water with soap and sanitizers at their workplace. It has been an established fact that the COVID-19 virus can live on surfaces for hours or possibly days.^14^ Disinfection of surfaces commonly touched by people including light switches, elevator buttons, door handles, countertops, chair arms, should be done on regular basis. This will also help in reassuring the anxious health care workers.

Care should be taken to avoid putting health care professionals under too much burden and making sure they are given enough time to rest, provision of food and other necessities. A study done in a hospital of Wuhan in China with relation to COVID-19 showed that increased exhaustion at work possibly increases the risk of being infected with COVID-19.^15^

One hundred forty-one (93.4%) participants were concerned about being exposed to COVID-19 at work. Choosing medical field as a profession, one knows the consequences they might face including high risk of exposure to contagious diseases. While health care professionals accept the responsibility to work in such an environment, they still have justified concern about the safety of their loved ones and family members at home. A recent study done in Pakistan showed 79.7 % doctors feared about infecting their family members during COVID-19 pandemic.^16^ In our study, 147 (97.4 %) participants had fear of taking the infection to loved ones at home.

Our study included participants from the public and private sector institutions in the city of Karachi, Pakistan. The findings do correlate with the presence of anxiety among health care professionals during a pandemic and certain factor playing a role in precipitating it. Due to rather new and early stages of this pandemic, there is insufficient data on similar published studies in Pakistan and hence comparison of different aspects of the results was rather difficult.

### Limitations of the Study

There are certain limitations to the study, such as the healthcare professionals include doctors, along with nursing staff and the paramedical staff, but only doctors were being considered in this study. Another limitation in this study is we did not assess previous psychiatric illness in the participants prior to COVID-19, the reason to do so is because psychiatric illness is still considered a social taboo in our society, even amongst the medical community, and to ask questions regarding such could have led to false information by the participants and no available centralized medical record data about individuals having anxiety to confirm any information.

## CONCLUSION

The study suggests that different grades of anxiety are present among doctors during pandemic COVID-19. Some contributory factors such as being female, healthcare workers dealing with suspected or confirmed cases may be associated with greater risk of having anxiety. Therefore, it is essential that the health care professionals are well taken care of not just physically but psychologically for them to be able to perform to their full potential. In doing so, certain guidelines and interventions are needed for the better care of the healthcare professionals.

### Author’s Contributions:

**SRH & ZH:** conceived the idea, contributed in writing the manuscript and are responsible for integrity of study.

**MTJ, RKA, ZH:** were involved in data collection.

**ZH:** was involved in data interpretation and statistical analysis.

All the authors approved the final version and are accountable.
